# The Fitting and Furnishing of Wards and Operating Theatres

**Published:** 1906-10-20

**Authors:** 


### THE FITTING AND FURNISHING OF WARDS AND OPERATING THEATRES.

### WALLS.

The principal functions of the walls of any hospital are to support the floors above it, and to exclude extremes of heat and cold. For both of these requirements strength is the prevailing factor. It goes without saying that a solidly-built wall is more satisfactory in every respect in the matter of support. There is less vibration, and all that follows in the wake of vibration. In addition also the strongly-built wall is a much better protection against the weather. For this purpose the wall that is built with an air space between its inner and outer portions, the air space being divided up by the bonds, offers the very best protection against damp, cold and heat. The air space helps very considerably to keep the inner wall dry, and it also offers the best-known resistance to the passage of heat through it. That is to say, in winter the ward which is so protected will be warmer when the biting northerly and easterly winds blow on its walls; and in summer will be cooler when the hot sun shines upon them, than a solid wall. The wall of the hospital ward also has the important office of carrying the windows. From the architect's point of view every window, every occasion that a wall is pierced weakens the wall by more than the actual depletion of the substance of which the wall is composed. In other words, it obliges the architect to make the remainder of the wall stronger than it otherwise would be, and this is particularly so in the case of hospital wards, where light is such an important feature. But after all mechanical requirements have been satisfied, aseptic requirements make very important demands upon the walls of wards, corridors, and operating theatres. As with the floors, there must be no harbour for germs. Naked brick or stone, even if otherwise suitable, would be *taboo*. They are porous, and nothing that is porous must occupy any space on walls, ceilings, or floors. Further, as with floors, there must be no corners, no angles or indentations of any kind where dust and other germs can settle, and all parts must be easily got at for thorough cleansing. Where the wall joins the floor it must be by a curve, and not by an angle.

At windows the recesses must curve, and not turn sharply. Obviously also the material of which the surfaces of the walls are composed must not crack, as every crack offers a

